# Early dysregulation of trigeminal motor pool excitability in a mouse model for neurodegenerative motoneuron disease

**DOI:** 10.1186/1471-2202-16-S1-P31

**Published:** 2015-12-18

**Authors:** Sharmila Venugopal, Martina Wiedau-Pazos, Scott H Chandler

**Affiliations:** 1 Department of Integrative Biology and Physiology, David Geffen School of Medicine, University of California Los Angeles, Los Angeles, CA, USA; 2 Department of Neurology, David Geffen School of Medicine, University of California Los Angeles, Los Angeles, CA, USA

## 

**Figure 1:**
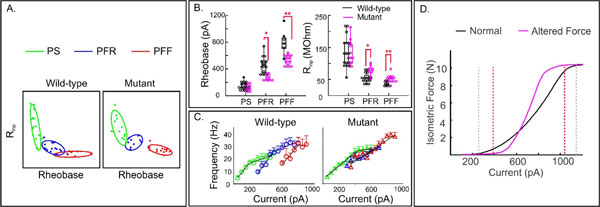
**A. Classification of TMNs into PFF, PFR and PS MU types using k-means clustering based on rheobase and input resistance (R_inp_)**. **B**. Rheobase values were significantly lower among mutant PFR and PFF units compared with wild-type (Student's t test, *p = 0.0002 for PFR, **p = 0.0001 for PFF), whereas R_inp _values were significantly greater (Student's t test, *p = 0.0009 for PFR, **p = 0.0028 for PFF). Error bars indicate SD. **C**. F-I relationships of PS, PFR and PFF TMNs in wild-type and mutant mice; color coding is similar to **A**. **D**. Simulation of isometric force versus MN input current across the jaw closer motor pool. Dashed lines demarcate normal (gray) and altered (red) muscle dynamic range.

Amyotrophic Lateral Sclerosis (ALS) is a progressive neurodegenerative motoneuron (MN) where in fast fatigable motor units (MUs) of vulnerable motor pools preferentially degenerate followed by fast fatigue resistant and slow MUs . Excitability is a key endogenous mechanism of MN neuroprotection [1] and therefore we hypothesize that pre-symptomatic excitability indicates impending disease development. Using a transgenic mouse model for ALS, we performed *in vitro *patch-clamp electrophysiology in ALS-vulnerable trigeminal motoneurons (TMNs) retrogradely labeled from jaw closer muscles at P8-12. We proposed a novel k-means clustering approach to classify TMNs into putative fast fatigable (PFF), fast fatigue resistant (PFR) and slow (PS) MUs based on rheobase and input resistance. Interestingly, hyper-excitability was noted in PFF and PFR TMNs (Fig. 1B) while hypo-excitability was evident in a subset of PS TMNs with linear frequency-current (F-I) characteristics compared to wild-type TMNs [2]. The F-I relationships displayed dysregulation across the motor pool (Fig. 1C). A jaw closer motor pool model was developed and simulated in MATLAB™ using observed alterations in membrane properties amongst the mutant MU types. Model results predict resistance in muscle force initiation and reduced motor pool dynamic range due to opposite changes in excitability between slow and fast TMNs (Fig. 1D).
